# Towards Specific Software Engineering Practices for Early-Stage Startups

**DOI:** 10.1007/978-3-030-58858-8_2

**Published:** 2020-08-18

**Authors:** Jorge Melegati, Rafael Chanin, Afonso Sales, Rafael Prikladnicki

**Affiliations:** 6 IT University of Copenhagen, Copenhagen, Denmark; 7 University of British Columbia, Vancouver, BC Canada; 8 Faculty of Computer Science, Free University of Bozen-Bolzano, Bolzano, Italy; 9 School of Technology, PUCRS, Porto Alegre, Brazil

**Keywords:** Early-stage startups, Innovation, Market-driven

## Abstract

In this position paper, our goal is to argue the need for specific software development practices to early-stage startups. In order to reach this goal, we discuss the consequences of innovative and market-driven contexts, which are two of the key elements when describing software startups. We also argue that these practices could be applied to innovative initiatives within established companies since they share similar characteristics and challenges as those from startups.

## Introduction

The definition of a startup is blurry in scientific research. There are two systematic mapping studies (SMS) performed on the topic, and both discussed how authors had defined the term. Back in 2014, Paternoster et al. [13] analyzed 43 primary studies and, as one of their results, grouped in themes the descriptions used by papers’ authors to characterize these companies. The list consisted of 15 themes where the most common were: 1. lack of resources; 2. highly reactive; 3. innovation; 4. uncertainty; 5. rapidly evolving; 6. time pressure.

In 2018, Berg et al. [3] repeated the analysis, including papers published in the period. They concluded that the rigor had increased, but there was not still a consensus on the term. However, in the period between the SMSs, the most common themes were innovation, uncertainty, small teams, lack of resources, and little or no operating history.

Startups follow a life-cycle composed of four stages: inception, stabilization, growth, and maturity [11]. Inception starts with the idea conception and ends with the first release. In the stabilization stage, the startup prepares to scale regarding technical and operational aspects. These two stages are the early-stages where the focus is on finding a relevant problem and solution. In the growth stage, the startup aims to reach the desired market participation, and, in the last stage, it progresses into an established company.

In a divisive paper, Klotins [10] argued that there is no characteristic unique to startups that are not observed in other teams developing innovative, market-driven, software-intensive products. To reach his conclusions, he reviewed the literature regarding themes identified by Paternoster et al. [13]. Below, we oppose this argument arguing that innovation and market-driven are necessary and sufficient elements to characterize startups. Still, this combination has slightly been touched in the software engineering literature. Finally, we show that current research to tackle problems in this context is still in its infancy and which avenues could be explored further.

**Fig. 1.**
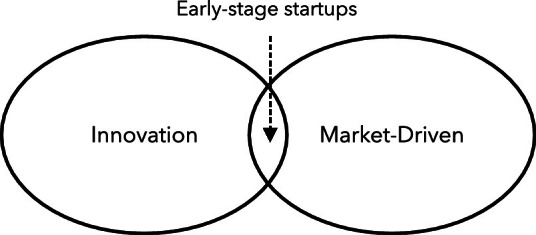
Early-stage startups: combining innovative and market-driven contexts.

## Necessity: Innovation and Market-Driven Context as a Challenge for Software Development in Startups

Innovation is an ambiguous term in the literature. To tackle this issue, Garcia and Calantone [7] reviewed studies on market, engineering, and new product development disciplines. The review showed that the term comprehends a discontinuity in marketing, technological, or both processes. In this context, ventures operate through several trials and errors along various dimensions of the business model [2]. This uncertain environment leads to challenges in software development like unstable requirements, compromised testing, and lack of written architecture specification [13]. These challenges exist, especially in market-driven contexts that are characterized by the software being developed to an open market with many customers instead of according to what is dictated by a paying customer (the so-called bespoke development).

A natural choice to deal with a dynamic context is to use agile methodologies since they embrace higher rates of change [15]. Nevertheless, agile methods may not be the final answer for software startups. Agile methods tackle changes through quick iterations with customer feedback [15]. However, these contact points are not available since, many times, even the customers are not known in the early-stages of a software startup. The lack of customer availability is a known challenge for teams applying these methods in market-driven environments [1, 9]. Therefore, the combination of innovation and a market-driven context leads to a situation where a specific set of practices would be useful. Figure 1 summarizes this argument.

## Sufficiency: Innovation on Software-Intensive Market-Driven Products as Startups

In this section, we argue that a team developing a new innovative, software-intensive, market-driven product is a software startup. Although this aspect contradicts common themes to describe software startups, such as lack of resources and lack of experience [13], teams in large companies formed to develop innovative products face similar problems as those from startups. Regardless of the context of the innovation process, uncertainty, time pressure, and the need to be highly reactive is always a part of the initiative. These characteristics require a particular way of tackling the idea being developed. A large organization cannot deal with uncertainty, for instance, just by adding more resources; the right approach needs to be implemented to transform the questions (or hypothesis) into facts.

To support our argument, we can mention the research on internal startups, in which teams develop innovative software-intensive products inside large companies. For instance, Edison et al. [5] investigated the use of Lean Startup in large companies, arguing that it facilitated the software product innovation in this context. That is, teams developing software-intensive innovative products, even in large companies, can use methods tailored to startups.

In this sense, we intend to formulate a set of best practices or a framework that could be applied in any scenario in which innovation on software-intensive market-driven products is being developed. We acknowledge that large organizations naturally differ from small ones. However, we can also find differences among small organizations: they may face different regulatory elements, competition, technical challenges, and so on. If the literature does not indicate that startups should apply different approaches depending on their characteristics, there is no reason not to include innovation software-intensive market-driven products or services being developed on large organizations.

## Current Proposals and Future Directions

Based on the arguments above, software startups would benefit from a set of practices tailored to an innovative process. Up to now, although a broad literature on the topic are being raised in the last years [3], there are no scientific studies proposing specific practices for these companies. Academic authors focused on describing the context including currently used practices (e.g., [8, 11, 12]) and faced challenges (e.g., [11, 14]).

Nevertheless, in the industry, some methodologies, like Lean Startup and Customer Development, are well-known. Although described based on anecdotal evidence and the authors’ own experience, several academics argued the influence and importance of experimentation to the core arguments of these practices, e.g., [4, 6]. Besides that, scientific studies in innovation and entrepreneurship literature have argued the value of experimentation in these contexts. Therefore, similar approaches seem a reasonable way to follow.

Our goal is to further explore our hypothesis by gathering data from initiatives in large organizations as well as from early-stage startups. By confirming our assumptions, we will work towards a set of software engineering practices for these teams. Of course, such endeavor is a huge challenge and, instead of a small team work, we expect this position paper acts as a call for the whole community to go towards this end. The literature described above will inform the creation of these practices.

## Conclusions

This position paper initiated a discussion on software engineering practices tailored to early-stage startups. Based on the fact that innovation and market-driven are usually used to define software startups, we argue that these aspects are decisive to characterize this context. Besides that, we claim that these aspects are also relevant to teams in other contexts, such as large companies. We hope that this discussion can encourage further investigation of specific practices for early-stage startups in any given context.

## References

[1] Ramesh B, Cao L, Baskerville R (2007). Agile requirements engineering practices and challenges: an empirical study. Inf. Syst. J..

[2] Andries P, Debackere K, van Looy B (2013). Simultaneous experimentation as a learning strategy: business model development under uncertainty. Strateg. Entrepreneurship J..

[3] Berg V, Birkeland J, Nguyen-Duc A, Pappas IO, Jaccheri L (2018). Software startup engineering: a systematic mapping study. J. Syst. Softw..

[4] Bortolini, R.F., Nogueira Cortimiglia, M., Danilevicz, A.d.M.F., Ghezzi, A.: Lean startup: a comprehensive historical review. Manag. Decis. (2018). 10.1108/MD-07-2017-0663

[5] Edison H, Smørsgård NM, Wang X, Abrahamsson P (2018). Lean internal startups for software product innovation in large companies: enablers and inhibitors. J. Syst. Softw..

[6] Frederiksen DL, Brem A (2016). How do entrepreneurs think they create value? A scientific reflection of Eric Ries’ Lean Startup approach. Int. Entrepreneurship Manag. J..

[7] Garcia R, Calantone R (2002). A critical look at technological innovation typology and innovativeness terminology: a literature review. J. Prod. Innov. Manage.

[8] Gralha, C., Damian, D., Wasserman, A.I.T., Goulão, M., Araújo, J.: The evolution of requirements practices in software startups. In: Proceedings of the 40th International Conference on Software Engineering - ICSE 2018, pp. 823–833. ACM Press, New York (2018). 10.1145/3180155.3180158

[9] Inayat I, Salim SS, Marczak S, Daneva M, Shamshirband S (2015). A systematic literature review on agile requirements engineering practices and challenges. Comput. Hum. Behav..

[10] Klotins, E.: Software start-ups through an empirical lens: are start-ups snowflakes?. In: CEUR Workshop Proceedings, vol. 2305, pp. 1–14 (2018)

[11] Klotins E (2019). A progression model of software engineering goals, challenges, and practices in start-ups. IEEE Trans. Software Eng..

[12] Melegati J, Goldman A, Kon F, Wang X (2019). A model of requirements engineering in software startups. Inf. Softw. Technol..

[13] Paternoster N, Giardino C, Unterkalmsteiner M, Gorschek T, Abrahamsson P (2014). Software development in startup companies: a systematic mapping study. Inf. Softw. Technol..

[14] Wang X, Edison H, Bajwa SS, Giardino C, Abrahamsson P, Sharp H, Hall T (2016). Key challenges in software startups across life cycle stages. Agile Processes, in Software Engineering, and Extreme Programming.

[15] Williams L, Cockburn A (2003). Agile software development: it’s about feedback and change. Computer.

